# Comparative molecular profiling of HPV‐induced squamous cell carcinomas

**DOI:** 10.1002/cam4.1108

**Published:** 2017-05-29

**Authors:** Robert F. Koncar, Rebecca Feldman, El Mustapha Bahassi, Nooshin Hashemi Sadraei

**Affiliations:** ^1^ Department of Internal Medicine Division of Hematology/Oncology University of Cincinnati Cincinnati Ohio; ^2^ Caris Life Sciences Phoenix Arizona

**Keywords:** Biomarkers, DNA sequencing, HPV, molecular profiling, protein expression, squamous cell carcinoma

## Abstract

Approximately 5% of all cancer incidences result from human papillomavirus (HPV) infection. HPV infection most commonly leads to cancers of the anogenital region or oropharynx. It is unknown whether different HPV‐mediated cancers collectively share a molecular signature and it is important to determine if there are targetable alterations common to different types of HPV‐positive tumors. We analyzed 743 p53 wild‐type samples of anal, cervical, oropharyngeal, and vulvar squamous cell carcinomas which underwent multiplatform testing at a commercial molecular profiling service. Expression of 24 proteins was measured by immunohistochemistry (IHC), mutation of 48 genes was determined by next‐generation and Sanger sequencing, and copy number alteration for six genes was determined by in situ hybridization. The four cohorts had remarkably similar molecular profiles. No gene had a statistically significant difference in mutation frequency or copy number change between the four different types of squamous cell carcinomas. The only significant differences between cohorts were frequency of ERCC1 and SPARC loss as determined by IHC. In all four cancer types, oncogene mutation and PD‐L1 expression was relatively infrequent. The most commonly mutated gene was *PIK3CA*, with mutations most often affecting the helical domain of the protein and accompanied by concurrent lack of PTEN expression. Loss of MGMT and RRM1 was common among the four cohorts and may be predictive of response to cytotoxic therapies not currently being used to treat these cancer types. The similar molecular profiles of the four cohorts indicate that treatment strategies may be similarly efficacious across HPV‐positive cancers.

## Introduction

Human papillomavirus (HPV) is responsible for nearly 1 out of every 20 cancer cases worldwide 1. HPV‐mediated cellular transformation is achieved primarily through expression of the viral E6 and E7 oncogenes. E6 and E7 act on a variety of targets to induce cell cycle progression. The most critical events for tumor development seem to be the E6‐mediated degradation of the tumor suppressor p53 and induction of telomerase expression, and the E7‐mediated degradation of the retinoblastoma (Rb) family of proteins 2, 3, 4, 5. p53 and Rb1 are important tumor suppressor proteins that play critical roles in restricting cell cycle progression and maintaining genomic stability. Consequently, E6 and E7 not only enhance cell proliferation, but also the acquisition of additional oncogenic mutations associated with a loss of genomic stability. While HPV‐negative squamous cell carcinomas (SCC) usually have p53 mutations, most HPV‐positive tumors are wild type for both p53 and Rb since both pathways are already inactivated by E6 and E7^6^, ^7^, ^8^, ^9^.

In the United States, Oropharyngeal (OSCC), Cervical (CSCC), Anal (ASCC), and Vulvar (VSCC) squamous cell carcinomas account for the largest number of new HPV‐associated cancers each year 10. Over 90% of CSCC and ASCC cases are caused by HPV and about 70% of OSCC and VSCC are the result of HPV infection 11. The management of CSCC, OSCC, ASCC, and VSCC often includes a multimodal treatment strategy that may include chemotherapy, radiation therapy, and surgery. Importantly, platinum‐based chemo‐radiation protocols are similarly effective against ASCC, OSCC, CSCC, and VSCC, suggesting different HPV‐positive cancers may have a common molecular signature and share the same targetable genetic alterations 12. Tailoring customized treatment regimens based on the molecular profiling of advanced solid tumors has been successful in several early phase trials 13, 14, 15. Therefore, we assessed CSCC, ASCC, OSCC, and VSCC for additional evidence of shared characteristics that could be used to identify potential molecular targets across the spectrum of HPV‐induced cancers.

In this study, we examined biomarker frequency data collected from a commercial molecular profiling service (Caris Life Sciences, Phoenix, AZ). Since p53 mutation is rare in HPV‐positive tumors, p53 status was used as a surrogate for HPV testing 16, 17. By analyzing p53 wild type‐SCC of the cervix, anal canal, oropharynx, and vulva, we sought to determine whether different HPV‐positive cancers may respond to common treatment protocols and to identify targetable alterations for patients with these cancers that are advanced, refractory, and difficult to treat. Using a multiplatform approach, this analysis identified several alterations that have the potential to aid in the design of treatment regimens. Our results revealed marked similarities among the HPV‐induced cancers indicating that similar treatment protocols may be beneficial to these cancer types. Importantly, this detailed examination of protein, genetic, and genomic alterations may provide insight for the design of future clinical trials.

## Methods

### Patients and multiplatform molecular profiling

Molecular theranostic biomarkers were assayed by immunohistochemistry, in situ hybridization, and gene sequencing. The biomarker‐drug associations are determined from prospective or retrospective clinical research studies in various solid tumors or are part of the National Comprehensive Cancer Network (NCCN) Biomarkers Compendium. For some biomarkers, therapeutic associations are suggested based on emerging data (e.g., investigational agents in clinical trials). This study includes data from 743 patients with refractory, aggressive, and/or metastatic ASCC, OSCC, CSCC, or VSCC with wild‐type p53. All samples were prospectively assayed by at least one platform (immunohistochemical (IHC), in situ hybridization (ISH), and Sanger/next‐generation sequencing (NGS)) by Caris Life Sciences (2009–2016).

Formalin‐fixed paraffin‐embedded (FFPE) samples were sent by treating physicians for biomarker analysis. Board‐certified pathologists verified sufficient tumor content, specimen quality, and confirmation of diagnosis for all tumors. The manner and scope of testing performed for each patient may have varied based on the physician's request, tissue availability, technology advancements (e.g., Sanger vs. NGS), and emerging clinical evidence for theranostic biomarkers.

### Immunohistochemistry

Immunohistochemistry (IHC) analysis of 24 proteins was performed by Caris Life Sciences on FFPE tumor samples using commercially available detection kits and automated staining techniques (Benchmark XT; Ventana Medical Systems, Tucson, AZ; and Autostainer‐ LInk 48; Dako, Carpinteria, CA) and manually scored by board‐certified pathologists. IHC analysis, antibody clones, and thresholds used are described in Table S1. IHC for p16 was performed by the Cincinnati Children's Hospital pathology core using antibody clone E6H4 (Ventana Medical Systems, Tucson, AZ) and positivity was determined by the authors.

### In situ hybridization

c‐MET, c‐MYC, EGFR, HER2, PIK3CA, and TOP2A gene copy number alterations were analyzed using fluorescence in situ hybridization (ISH) and/or chromogenic ISH probes as part of the automated staining techniques (Benchmark XT; Ventana Medical Systems) and automated imaging systems (BioView, Billerica, MA). Copy number alteration was determined as previously described 9.

### Sanger sequencing

Sanger sequencing included selected regions of *BRAF*,* c‐KIT, EGFR, KRAS, NRAS*, and *PIK3CA* and was performed using M13‐linked polymerase chain reaction primers designed to flank and amplify targeted sequences. Polymerase chain reaction products were bidirectionally sequenced using the BigDye Terminator version 1.1 chemistry, and analyzed using the 3730 DNA Analyzer (Applied Biosystems, Grand Island, NY). Sequence traces were analyzed using Mutation Surveyor software version 3.25 (Soft Genetics, State College, PA).

### Next‐generation sequencing

NGS was performed on gDNA from FFPE tumor tissue using the Illumina MiSeq. Specific regions of 48 genes were amplified with the Illumina TruSeq Amplicon Cancer Hotspot panel. All reported mutations were detected with over 99% confidence with an average sequencing depth of over 1000×.

### Statistical methods

Retrospective analysis of biomarker frequency was determined using standard descriptive statistics. The two‐tailed Fisher's exact test was performed using JMP version 10.0 (SAS Institute, Cary, NC) to test where frequencies differed among cohorts. Bonferroni correction was used to correct for multiple comparisons. A corrected two‐tailed *P* value ≤ 0.05 was determined statistically significant.

### Validation and institutional review board

All methods utilized in this study were clinically validated to Clinical Laboratory Improvement Amendments, College of American Pathologists, and International Organization for Standardization (ISO) 15,189. This retrospective analysis utilized previously collected, de‐identified data created under the Caris honestbroker policy and followed consultation with the Western Institutional Review Board (IRB), which is the IRB of record for Caris Life Sciences. The project was determined to be exempt from IRB oversight and consent requirements were waived.

## Results

### Patient and Tumor characteristics

ASCC, CSCC, OSCC, and VSCC tumors were screened for p53 status as a surrogate for HPV testing. A total of 743 tumors with wild‐type p53 status were identified for this study and are described in Table 1. While over 90% of all ASCC and CSCC cases result from HPV infection, only 70% of OSCC cases are HPV positive and tobacco or alcohol use likely contribute to the other 30% 11, 18. To further validate that the cohorts contained HPV‐positive samples, OSCC tissue was also tested for p16 immunoreactivity, which is a reliable, clinically utilized indicator of HPV positivity 19. Of the 147 p53 wild‐type OSCC specimens, 128 had slides of tissue available for p16 IHC. Over 97% of the OSCC samples tested (125/128) were positive for p16 expression (Table S2). CSCC samples accounted for approximately 42% (*n* = 314) of all tumors. ASCC, OSCC, and VSCC accounted for 27% (*n* = 199), 20% (*n* = 147), and 11% (*n* = 83) of samples, respectively. The ASCC cohort was predominantly female (64%), while the male sex made up the majority of the OSCC cohort (84%). Of the 743 tumor samples, 337 (45%) were from primary tumors and 406 (55%) were metastatic.

**Table 1:** Clinicopathological characteristics of 743 patients with molecularly profiled p53 wild‐type squamous cell carcinoma

	ASCC	CSCC	OSCC	VSCC
Male (%)	70 (35.2%)	0 (0%)	124 (84.4%)	0 (0%)
Average age, years	59	N/A	58	N/A
Minimum age	31	N/A	27	N/A
Maximum age	88	N/A	88	N/A
Site of tumor profiled (%)				
Primary site	28 (40%)	N/A	67 (54%)	N/A
Metastatic site	42 (60%)	N/A	57 (46%)	N/A
Female (%)	129 (64.8%)	314 (100%)	23 (15.6%)	83 (100%)
Average age, years	59	50	61	61
Minimum age	35	24	26	29
Maximum age	89	90	79	85
Site of tumor profiled (%)				
Primary site	36 (27.9%)	141 (45%)	15 (65.2%)	50 (60.2%)
Metastatic site	93 (72.1%)	173 (55%)	8 (34.8%)	33 (39.8%)
Total	199	314	147	83
Site of tumor profiled (%)				
Primary site	64 (32.2%)	141 (45%)	82 (55.8%)	50 (60.2%)
Metastatic site	135 (67.8%)	173 (55%)	65 (44.2%)	33 (39.8%)

### Multiplatform biomarker detection

**Figure 1:**
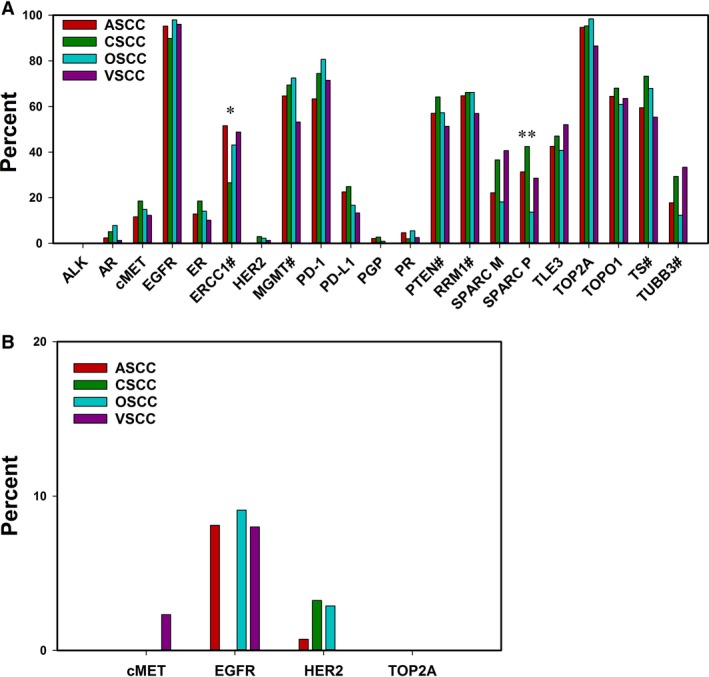
HPV‐positive squamous cell carcinomas have similar rates of expression profile alteration. (A) IHC revealed similar rates of biomarker detection in ASCC, CSCC, OSCC, and VSCC tumors. Positivity is indicative of high expression unless denoted. # indicates low or no expression as positive. (B) Frequency of gene amplification across SCCs as determined by ISH. *P* < 0.05 (*); *P* < 0.001 (**).

A total of 79 biomarkers were evaluated in this study using a combination of next‐generation (NGS) and Sanger sequencing, Immunohistochemistry (IHC), and in situ hybridization (ISH). However, due to variation in the number of biomarkers tested for each sample, the total number of patients assayed differs for each biomarker. The total numbers of patients tested for each biomarker are listed in Tables 2 and 3. Only biomarkers that had been tested in all four types of SCC were included in further analysis. Therefore, IHC testing of BCRP, c‐kit, COX‐2, and MRP1 as well as ISH testing of *c‐MYC* and *PIK3CA* were not compared across types of SCC since there were no data from one or more SCC cohorts (Fig. 1).

**Table 2:** Alteration frequency of predictive biomarkers measured by immunohistochemistry and in situ hybridization

Method	Biomarker	Number of samples tested	Number of samples altered	% altered
IHC (high expression)	ALK	123	0	0.0
	AR	648	29	4.5
	BCRP	9	1	11.1
	c‐kit	104	2	1.9
	cMET	538	84	15.6
	COX‐2	10	3	30.0
	EGFR	273	254	93.0
	ER	680	104	15.3
	HER2	700	13	1.9
	MRP1	102	91	89.2
	PD‐1	326	239	73.3
	PD‐L1 (SP142)	368	81	22.0
	PGP	588	11	1.9
	PR	677	23	3.4
	RRM1	596	386	64.8
	SPARC M	506	146	28.9
	SPARC P	546	170	31.1
	TLE3	548	248	45.3
	TOP2A	661	626	94.7
	TOPO1	679	443	65.2
IHC (low/no expression)	ERCC1	296	122	41.2
	MGMT	669	447	66.8
	PTEN	694	412	59.4
	TS	690	460	66.7
	TUBB3	571	138	24.2
ISH (amplification)	c‐MET	421	1	0.2
	c‐MYC	4	1	25.0
	EGFR	85	7	8.2
	HER2	574	13	2.3
	PIK3CA	3	3	100.0
	TOP2A	33	0	0.0

**Table 3:** Mutation frequency as determined by next‐generation sequencing

Biomarker	Number tested	Number altered	% altered
ABL1	442	0	0.0
AKT1	451	9	2.0
ALK	453	0	0.0
APC	452	1	0.2
ATM	442	1	0.2
BRAF	537	0	0.0
BRCA1	221	2	0.9
BRCA2	222	3	1.4
CDH1	452	0	0.0
c‐KIT	507	0	0.0
cMET	453	0	0.0
CSF1R	451	0	0.0
CTNNB1	453	8	1.8
EGFR	458	0	0.0
ERBB2	448	0	0.0
ERBB4	449	0	0.0
FBXW7	450	29	6.4
FGFR1	453	0	0.0
FGFR2	452	0	0.0
FLT3	452	0	0.0
GNA11	403	0	0.0
GNAQ	350	0	0.0
GNAS	453	1	0.2
HNF1A	408	2	0.5
HRAS	373	3	0.8
IDH1	452	0	0.0
JAK2	453	0	0.0
JAK3	452	0	0.0
KDR	453	0	0.0
KRAS	567	11	1.9
MLH1	451	0	0.0
MPL	449	0	0.0
NOTCH1	448	0	0.0
NPM1	451	0	0.0
NRAS	502	2	0.4
PDGFRA	451	0	0.0
PIK3CA	527	136	25.8
PTEN	432	14	3.2
PTPN11	452	0	0.0
RB1	445	3	0.7
RET	442	0	0.0
SMAD4	452	1	0.2
SMARCB1	451	0	0.0
SMO	361	0	0.0
STK11	434	2	0.5
TP53	446	0	0.0
VHL	405	0	0.0

### Gene expression and copy number alteration

Total rates of gene expression as measured by IHC and gene copy number alteration by ISH are listed in Table 2. IHC revealed the most frequently altered proteins to be: TOP2A (94.7%), EGFR (93%), MRP1 (89.2%), and PD‐1 (73.3%), which were all highly expressed. Loss of MGMT (66.8%), TS (66.7%), and PTEN (59.4%) expression also occurred at high frequency. ISH showed the *PIK3CA* and *c‐MYC* genes were amplified in 100% and 25% of tested samples, respectively. However, only three patients were tested for *PIK3CA* and only four patients were tested for *c‐MYC* amplification. No other amplifications were detected at high rates. The only other amplifications detected were *EGFR, HER2*, and *c‐MET* which occurred in less than 10% of tested samples.

Markedly similar expression profiles were detected between ASCC, CSCC, OSCC, and VSCC (Fig. 1A). In all four cohorts, IHC most commonly revealed high expression of EGFR, which ranged from detection rates of 89.8% in CSCC to 97.9% in OSCC, and TOP2A, which ranged from detection rates of 86.5% in VSCC to 98.4% in OSCC. While EGFR detection was common, ALK expression was not detected in any of the cohorts. The biomarker with the largest variance of detection among the four types of SCC was SPARC P. Over 42% of CSCC samples were positive for SPARC P (*P* < 0.001), while only 13.7% of OSCC samples were positive (*P* < 0.001).

ERCC1 expression was lost in over half of ASCC samples and over 43% of OSCC and VSCC samples. However, only 26.5% of CSCC samples lost ERCC1 expression (*P* = 0.024). MRP1 was detected at high frequency in ASCC (97.7%), OSCC (86.2%), and VSCC (79.3%) though no cases of CSCC were tested for MRP1 by IHC. The detection rates of RRM1 and TLE3 expression were also consistent across cohorts and ranged from 56% to 66% and 42% to 52%, respectively. Loss of PTEN, MGMT, and TS expression was also consistent among all four groups. Rates of PTEN loss ranged from 51.2% in VSCC to 64.1% in CSCC. MGMT and TS loss rates ranged from 53% to 73%.

The PD‐1/PD‐L1 immunomodulatory axis was also evaluated across the four cohorts of SCC. Tumor infiltrating lymphocytes were examined for PD‐1 expression and tumor cells were tested for PD‐L1 expression. Positivity for PD‐1 was more common than for PD‐L1. Rates of PD‐1 expression positivity ranged from 63.3% in ASCC to 80.6% in OSCC, while PD‐ L1 expression positivity rates ranged from only 13.3% in VSCC to 24.9% in CSCC.

Only c‐MET, EGFR, HER2, and TOP2A were examined for gene copy number alterations in samples from all four cohorts. Gene amplification was found by ISH to be an uncommon event, regardless of cancer type (Fig. 1B). The most commonly amplified gene was *EGFR* which was amplified in 9.1% of OSCC, 8.1% of ASCC, 8% of VSCC, and 0% of CSCC cases. Amplification of *c‐MET* was detected only in a single case of VSCC (2.3%), while *TOP2A* amplification was not found in any samples. *PIK3CA* amplification was examined in only two cases of ASCC and one case of OSCC, all of which tested positive, while *c‐MYC* copy number increase was detected in one of three tested OSCC samples and was not amplified in the one ASCC sample tested.

### Mutation analysis

Total rates of variant detection for the 47 genes analyzed for mutation are shown in Table 3. Specific allele changes are listed in Table S3 and mutation profiles by patient are listed in Table S4. The most frequently mutated gene was *PIK3CA* which was altered in 25.8% of SCC patients tested. The next most commonly mutated genes were *FBXW7* (6.4%) and *PTEN* (3.2%). No known oncogenic mutations were detected in the following genes in any of the four cohorts: *ABL1, ALK, BRAF, CDH1, c‐KIT, c‐MET, CSF1R, EGFR, ERBB2, ERBB4, FGFR1, FGFR2, FLT3, GNA11, GNAQ, IDH1, JAK2, JAK3, KDR, MLH1, MPL, NOTCH1, NPM1, PDGFRA, PTPN11, RET, SMARCB1, SMO*, and *VHL* (Table 3).

**Figure 2:**
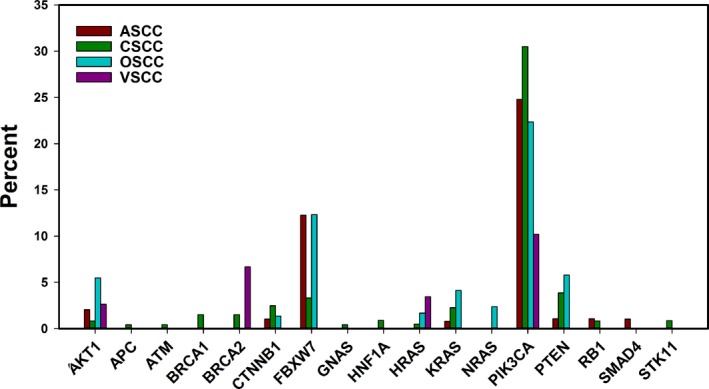
Mutation of biomarker genes occurs in similar proportions of different HPV+ squamous cell carcinomas. Mutations of clinical significance were identified through sequencing of 47 genes. Genes that were not mutated in any of the four SCC types were not included in this figure. No difference between cohorts was statistically significant.

*PIK3CA* was the most commonly mutated gene in all four types of SCC, occurring at a rate of 24.8% in ASCC, 30.5% in CSCC, 22.3% in OSCC, and 10.2% in VSCC (Fig. 2). Other than *PIK3CA* mutation, mutational events were relatively uncommon in all four cohorts. The next most commonly mutated gene in ASCC and OSCC was *FBXW7* (12%). However, only 3% of CSCC samples were mutant and no *FBXW7* mutations were detected in VSCC samples. The second most common mutation in VSCC was *BRCA2* (6.7%), which was not detected in ASCC or OSCC and found in only 1.5% of CSCC cases. The only gene found mutated within all four cohorts, aside from *PIK3CA*, was *AKT1*, albeit at a very low rate (0.8%‐5.5%). No differences in mutation rates were statistically significant between the four cohorts.

### PI3K pathway

Phosphoinositide 3‐kinase (PI3K) is a heterodimer lipid kinase that, when activated by a receptor tyrosine kinase, converts PIP_2_ to PIP_3_ 20. The p110*α* catalytic subunit of the enzyme is encoded by the *PIK3CA* gene. PIP_3_ recruits AKT to the membrane, where it can be activated and promote proliferation, growth, and survival through activation of downstream targets such as mTOR. The phosphatase PTEN acts antagonistically to PI3K, dephosphorylating PIP_3_ to PIP_2_ and preventing AKT recruitment and activation 21, 22.

**Figure 3:**
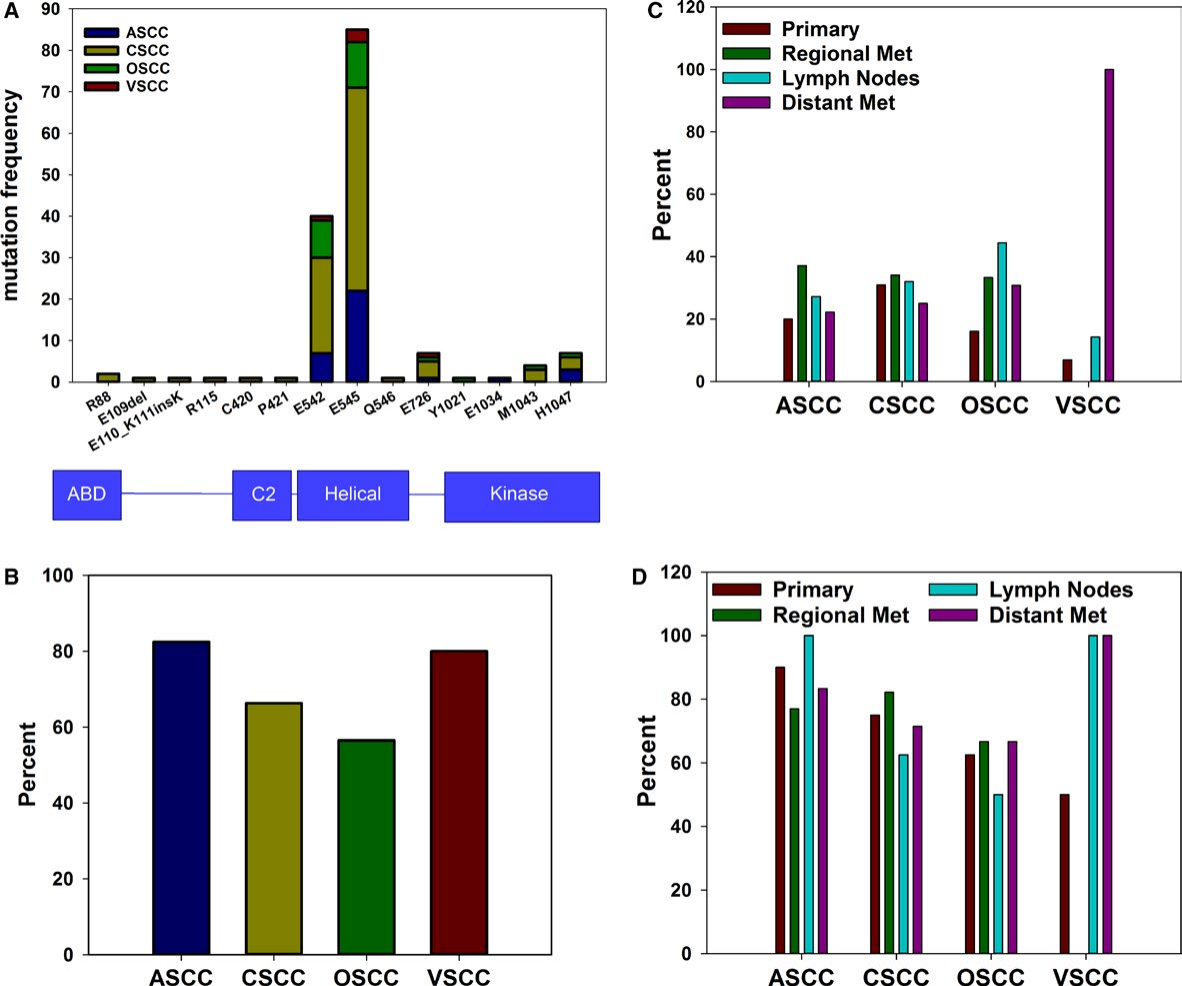
Different HPV+ squamous cell carcinomas have similar PIK3CA mutational landscapes. (A) Frequency and distribution of mutations within the PIK3CA gene as determined by NGS and Sanger sequencing. Corresponding protein domains are shown below the graph. (B) Percent of PIK3CA‐mutant tumors with co‐occurring loss of PTEN as determined by IHC. (C) Percent of samples with PIK3CA mutation stratified by disease state. For primary tumors, *n* = 50 (ASCC), *n* = 113 (CSCC), *n* = 56 (OSCC), *n* = 29 (VSCC). For regional metastases, *n* = 35 (ASCC), *n* = 85 (CSCC), *n* = 12 (OSCC), *n* = 3 (VSCC). For samples from lymph nodes, *n* = 11 (ASCC), *n* = 25 (CSCC), *n* = 9 (OSCC), *n* = 14 (VSCC). For distant metastases, *n* = 27 (ASCC), *n* = 32 (CSCC), *n* = 13 (OSCC), *n* = 1 (VSCC). (D) Percent of PIK3CA‐mutant samples with co‐occurring loss of PTEN expression. For primary tumors, *n* = 10 (ASCC), *n* = 32 (CSCC), *n* = 8 (OSCC), *n* = 2 (VSCC). For regional metastases, *n* = 13 (ASCC), *n* = 28 (CSCC), *n* = 3 (OSCC), *n* = 0 (VSCC). For samples from lymph nodes, *n* = 2 (ASCC), *n* = 8 (CSCC), *n* = 4 (OSCC), *n* = 2 (VSCC). For distant metastases, *n* = 6 (ASCC), *n* = 7 (CSCC), *n* = 3 (OSCC), *n* = 1 (VSCC). No differences were statistically significant after correcting for multiple comparisons.

The specific site of a *PIK3CA* mutation is potentially an important predictive indicator for tumor growth and response to treatment. In urothelial carcinoma, mutations in the kinase domain of the protein result in increased AKT activation, compared to helical domain mutations 23. Additionally, specific *PIK3CA* mutations may have implications for treatment efficacy. Patients with advanced solid tumors harboring an H1047R mutation, which is in the kinase domain, benefit from treatment with a PI3K/AKT/mTOR inhibitor more than patients with other *PIK3CA* mutations 24 Examination of specific *PIK3CA* mutation loci revealed that all four types of SCC had similar distributions of mutation sites within the *PIK3CA* gene (Fig. 3A). In each of the four cohorts, E545 was the most commonly mutated site and a missense mutation at either E542 or E545 in the helical domain of the protein accounted for 75% of all *PIK3CA* mutations. Mutations in the kinase domain were not common as they accounted for 12% of the *PIK3CA* mutations for OSCC and ASCC. In CSCC, only 7% of *PIK3CA* mutations were in the kinase domain and none were detected in VSCC. However, there were only five cases of *PIK3CA*‐mutant VSCC in the study. Interestingly, mutation of E726 in exon 13, which is of unknown clinical significance, was detected in all four cohorts.

PI3 kinase inhibitors are currently in clinical trials and loss of PTEN expression has emerged as an indicator of resistance to PI3K inhibition 25. It was previously reported that 24% of head and neck SCC with a PI3K mutation also exhibit loss of PTEN expression 9. HPV‐positive tumors exhibited much higher rates of co‐occurrence in all four cohorts (Fig. 3B). In *PIK3CA*‐mutant samples, concurrent PTEN loss was found in 82.4% (ASCC), 80% (VSCC), 66.3% (CSCC), and 56.5% (OSCC) of cases.

*PIK3CA* mutation status and PTEN expression was then examined based on disease state for each of the four SCCs. Disease state was classified depending upon whether the sample was collected from the primary tumor, regional metastasis into the surrounding soft tissue, lymph node, or distant metastasis. *PIK3CA* mutation rate in ASCC ranged from 20% in specimens from the primary tumor site to 37% in samples from regional metastases (Fig. 3C). The mutation rate in CSCC ranged from 25% in distant metastases to 34% in regional metastases. OSCC mutation rates were lowest in tissue from the primary tumors (16%) and highest in samples collected from the lymph nodes (44%). VSCC had the widest range of *PIK3CA* mutation rates with no mutations detected in regional metastases, while the only sample from a case of distant metastasis was positive for *PIK3CA* mutation. Only 47 VSCC samples could be included for comparison of *PIK3CA* mutation by disease state and of these, only three were of regional metastases. No differences were statistically significant after correction for multiple comparisons.

*PIK3CA*‐mutant samples stratified by disease state were also examined for concurrent loss of PTEN (Fig. 3D). In cases of *PIK3CA*‐mutant ASCC, PTEN loss was detected in 77% of samples from regional metastases, 83% of distant metastases, 90% of primary tumors, and 100% of samples from lymph nodes. For CSCC, *PIK3CA*‐mutant samples from lymph nodes had the lowest rate of PTEN loss (63%), while samples from regional metastases had the highest (82%). OSCC samples ranged from 50% in lymph node samples to 67% in samples from regional and distant metastases. *PIK3CA*‐mutation was not detected in any VSCC samples of regional metastases; so, concurrent PTEN loss could not be examined. Rates of PTEN loss ranged from 50% in primary tumor samples to 100% in samples from lymph nodes and distant metastases. Despite the wide range of PTEN loss rates in VSCC, there were only five total *PIK3CA*‐mutant samples and no differences were statistically significant.

### PD‐1/PD‐L1

**Figure 4:**
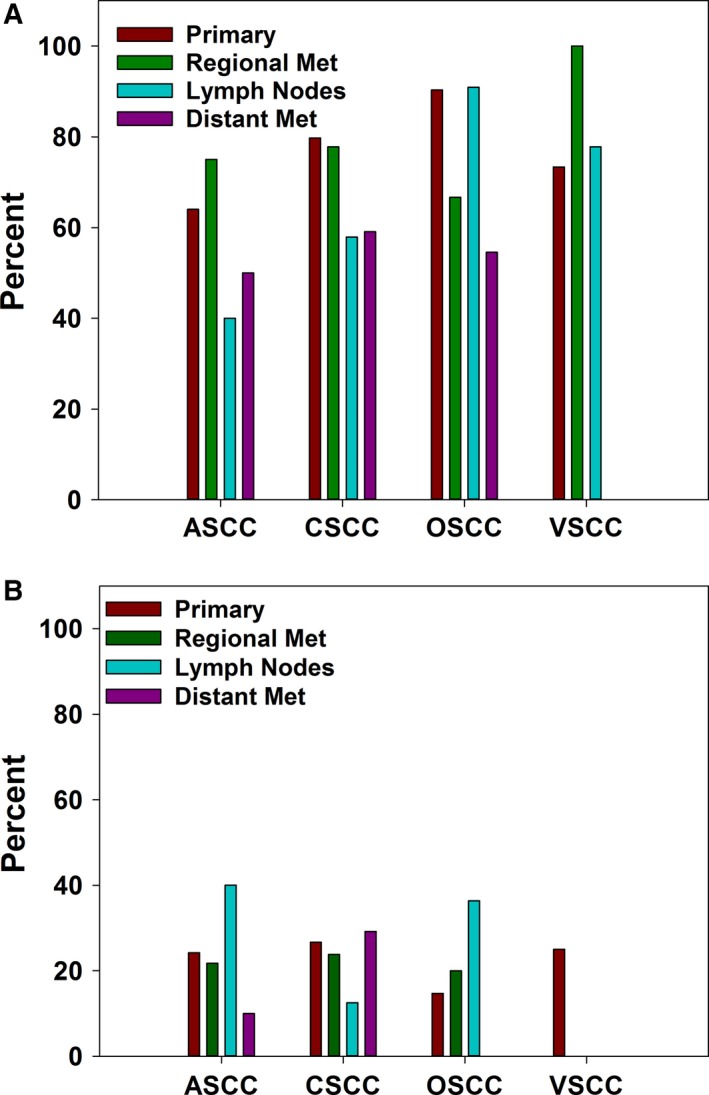
PD‐1 and PD‐L1 expression in different squamous cell carcinomas stratified by disease state. (A) Percent of samples positive for PD‐1 expression on tumor infiltrating lymphocytes as determined by IHC. For primary tumors, *n* = 25 (ASCC), *n* = 79 (CSCC), *n* = 31 (OSCC), *n* = 15 (VSCC). For regional metastases, *n* = 20 (ASCC), *n* = 54 (CSCC), *n* = 9 (OSCC), *n* = 2 (VSCC). For samples from lymph nodes, *n* = 5 (ASCC), *n* = 19 (CSCC), *n* = 11 (OSCC), *n* = 9 (VSCC). For distant metastases, *n* = 10 (ASCC), *n* = 22 (CSCC), *n* = 11 (OSCC), *n* = 2 (VSCC). (B) Percent of tumor samples positive for PD‐L1 expression by IHC. For primary tumors, *n* = 33 (ASCC), *n* = 90 (CSCC), *n* = 34 (OSCC), *n* = 16 (VSCC). For regional metastases, *n* = 23 (ASCC), *n* = 63 (CSCC), *n* = 10 (OSCC), *n* = 3 (VSCC). For samples from lymph nodes, *n* = 5 (ASCC), *n* = 24 (CSCC), *n* = 11 (OSCC), *n* = 9 (VSCC). For distant metastases, *n* = 10 (ASCC), *n* = 24 (CSCC), *n* = 11 (OSCC), *n* = 2 (VSCC).

With the emergence of anti‐PD‐1 therapies, PD‐L1 has become an important biomarker in an attempt to predict what patients would benefit from anti‐PD‐1 treatments 26. In all four cohorts, PD‐1 expression on tumor infiltrating lymphocytes (TIL) was much more common than PD‐L1 expression on tumor cells (Fig. 1). PD‐1 and PD‐L1 expression were analyzed by disease state for each type of SCC (Fig. 4A, B). In ASCC, PD‐1 positivity on TIL ranged from 40% in samples from lymph nodes to 75% in regional metastases. For CSCC, PD‐1 positivity was more common in primary tumors (79.7%) and regional metastases (77.8%) than in samples from lymph nodes (57.8%) and distant metastases (59.1%). Over 90% of OSCC samples from primary tumors and lymph nodes were positive for TIL expression of PD‐1. Positivity rates for regional and distant metastases were 66.7% and 54.5%, respectively. Two out of two VSCC samples from regional metastases tested for PD‐1were positive for expression on TIL, while neither of the two samples tested from distant metastases were positive. VSCC samples from primary tumors (73.3%) and lymph nodes (77.8%) were similar in terms of PD‐1 expression. No differences in PD‐1 expression were statistically significant. Additionally, there was not a statistically significant difference in PD‐1 expression between primary and metastatic tumors in general (Fig. S1).

Tumor cell expression of PD‐L1 was also tested. Within primary tumors and regional metastases, PD‐L1 expression was similar in the different types of SCC. In primary tumors, PD‐L1 expression was most common in CSCC (26.7%) and least common in OSCC (14.7%). In regional metastases, ASCC, CSCC, and OSCC positivity rates ranged from 20% (OSCC) to 23.9%. No VSCC samples of regional metastases were positive for PD‐L1 expression, though only three samples were tested. None of the nine VSCC samples from lymph nodes or two samples from distant metastases were positive for PD‐L1 expression either. Rates of positivity within lymph node samples were 40% (ASCC), 12.5% (CSCC), and 36.4% (OSCC). For distant metastases, none of the 11 OSCC samples tested were positive for PD‐L1 expression. ASCC and CSCC samples had positivity rates of 10% and 29.1%, respectively. No differences in rates of PD‐L1 expression were statistically significant.

## Discussion

All four types of SCC showed a strikingly similar molecular profile. Chemotherapeutic treatment options are similar for ASCC, CSCC, OSCC, and VSCC with 5‐fluorouracil and platinum‐based therapies commonly used across all four types of cancer. However, CSCC currently has more treatment options than the other three types of SCC evaluated (Table 4). The similarities between the four cohorts suggest that treatments shown to be effective in HPV‐positive CSCC may also be beneficial in the treatment of ASCC, OSCC, and VSCC. Low RRM1 expression is used to predict gemcitabine efficacy in CSCC (Table 4) and is common in all four cohorts (Fig. 1A), suggesting gemcitabine may be beneficial beyond just CSCC. Taxanes are currently used to treat CSCC, OSCC, and VSCC and are currently in clinical trials for use in ASCC, where the results are very promising, particularly in HPV‐positive cases 27.

**Table 4:** Comparison of cancer agents according to NCCN guidelines for HPV‐induced cancers

Drug Type	Predictive Biomarkers	ASCC	CSCC	OSCC	VSCC
Cytotoxic Therapies	ERCC1, BRCA1/2	cisplatin	cisplatin, carboplatin	cisplatin, carboplatin	cisplatin
	TS	5‐fluorouracil, capecitabine	5‐fluorouracil, pemetrexed	5‐fluorouracil, capecitabine	5‐fluorouracil
	TUBB3/TLE3/PGP		paclitaxel, docetaxel, vinorelbine	paclitaxel, docetaxel	paclitaxel, vinorelbine
	TOPO1		topotecan, irinotecan		
	RRM1		gemcitabine		
	BRCA1/2	mitomycin‐C	mitomycin‐C		mitomycin‐C
Targeted Therapies	none identified		bevacizumab	cetuximab	

In addition to cytotoxic agents, targeted therapies such as the anti‐EGFR antibody, cetuximab, are also being used. However, there are currently no recognized biomarkers to predict which patients will benefit from these treatments 28 (Table 4). While, *EGFR* amplification was not common, the vast majority of samples in all four cohorts highly expressed EGFR. Surprisingly, *EGFR* copy number increase is not predictive of cetuximab efficacy 29. In non‐small‐cell lung cancer, EGFR expression level is predictive of cetuximab benefit; however, for head and neck SCC and colon cancer, even EGFR expression level is not informative in determining if cetuximab will improve patient outcome 30, 31. Rather, evaluation of downstream signaling components (e.g., members of PI3K pathway) or other receptor tyrosine kinases (e.g., c‐MET, ROS1, ALK) seems to be more informative in predicting efficacy of anti‐EGFR therapy 32, 33, 34, 35, 36, 37. More work is needed to determine what molecular signatures are indicative of a positive response to anti‐EGFR treatments.

The rate of EGFR IHC positivity in our OSCC cohort is higher than reported in other HPV‐positive OSCC cohorts 38, 39, 40. This is likely because our data are generated from samples submitted to a commercial molecular profiling service. Samples from tumors that respond well to chemotherapy are unlikely to be submitted for molecular profiling and consequently, our cohort may contain a disproportionately high number of aggressive, treatment‐resistant tumors that have already been treated with chemotherapy. EGFR expression is associated with a poor prognosis in OSCC 38, further suggesting that our data describe the molecular profile of aggressive, treatment‐resistant HPV‐positive SCC, rather that HPV‐positive SCC in general.

IHC positivity was even more common for TOP2a than for EGFR. In hepatocellular carcinoma, TOP2a overexpression is associated with chemoresistance. However, there is some evidence that etoposide may be beneficial to these patients in combination with doxorubicin, another topoisomerase II inhibitor 41. In a mouse model of lymphoma, high TOP2a expression is an indicator of doxorubicin efficacy, while low TOP1 expression is associated with resistance to the topoisomerase I inhibitor camptothecin and increased responsiveness to doxorubicin 42. Multiple studies have found that TOP1 expression level is predictive of whether colon cancer patients will benefit from irinotecan 43, 44 and possibly predictive of oxaliplatin efficacy 43. Interestingly, while TOP1 expression level has shown great predictive value, *TOP1* copy number was not found to be a predictive biomarker for irinotecan 44. There have been several reports of using irinotecan and cetuximab as a successful combination therapy for ASCC, particularly in *KRAS* wild‐type patients 45, 46. These reports are in agreement with our finding that the majority of ASCC patients have high expression of both EGFR and TOP1, while also highlighting the potential importance of using biomarkers downstream of EGFR to predict the benefits of cetuximab. It remains to be seen if treatment regimens targeting EGFR and topoisomerase I are effective in CSCC, VSCC, and OSCC, but the similarities we have found between the four cohorts provides rationale for further investigation.

While we found TOP2a to be highly expressed in almost all cases of CSCC, in a trial of CSCC patients, orally administered etoposide as a second‐line therapy was only effective in 11% of patients 47, indicating that TOP2a expression status alone may not be predictive of response to topoisomerase II poisons. This is not unexpected considering that most patients in this study had high expression of both TOP2a and TOP1.

In hepatocellular carcinoma, TOP2a overexpression is associated with chemoresistance. However, there is some evidence that etoposide may be beneficial to these patients in combination with doxorubicin, another topoisomerase II inhibitor 41. In a mouse model of lymphoma, high TOP2a expression is an indicator of doxorubicin efficacy, while low TOP1 expression is associated with resistance to the topoisomerase I inhibitor camptothecin and increased responsiveness to doxorubicin 42. Multiple studies have found that TOP1 expression level is predictive of whether a colon cancer patient will benefit from irinotecan 43, 44 and possibly predictive of oxaliplatin efficacy 43. Interestingly, while TOP1 expression level has shown great predictive value, *TOP1* copy number was not found to be a predictive biomarker for irinotecan 44.

High expression of the nucleotide excision repair and DNA interstrand crosslink repair protein, ERCC1, is an established marker for resistance to platinum‐based chemotherapy 48, 49, 50. Over 40% of samples in this study displayed low ERCC1 expression. ERCC1 also seems to be necessary for homologous recombination, suggesting PARP inhibitors may provide additional therapeutic benefit to these patients 51. In vitro work suggests that the addition of a PARP inhibitor to a platinum‐based treatment strategy may be beneficial when tumors have low ERCC1 expression 52.

The majority of samples in all four cohorts displayed low MGMT expression. Loss of MGMT expression is an established indicator of response to the DNA methylating agent, temozolomide. While previous studies found that temozolomide offers little value outside of treating brain tumors, most studies did not select patients based on MGMT status 53. The use of MGMT as a biomarker for determining temozolomide utility outside of brain tumors has shown promise in case reports of colorectal cancer patients with low MGMT expression 54. Further investigation is needed to determine if MGMT is a useful indicator of response to temozolomide in HPV‐positive SCC.

Tumors expressing PD‐L1 are thought to have a better chance of responding to anti‐PD‐1 therapy, while the value of PD‐1 expression is less clear. Additional factors may play into determining the benefit of anti‐PD‐1 therapy, since some patients testing negative for PD‐L1 have benefited greatly from immune checkpoint inhibitors 26, 55, 56, 57, 58. Additional work is needed to determine the predictive value of PD1 and PD‐L1, specifically in HPV‐induced SCC. Immunotherapies may be particularly important for treating this type of cancer since there are few targeted therapies available as options for these patients (Table 4). Furthermore, most PIK3CA mutations in these tumors occur outside of the kinase domain and are accompanied by concurrent loss of PTEN expression (Fig. 3), making these tumors potentially poor responders to PI3K inhibitors and further highlighting the importance of determining the predictive value of PD‐1 and PD‐L1 with regard to anti‐PD1 therapy.

The similar molecular profiles of the four cohorts indicate that treatment strategies may be similarly efficacious across HPV‐positive cancers. The low rate of oncogene mutation detected in the tumor samples suggests that there is limited potential for targeted therapies in HPV‐positive SCC and emphasizes the importance of identifying reliable biomarkers for current and future cytotoxic and immunotherapies.

## Conflict of Interest

Rebecca Feldman is employed by Caris Life Sciences as a research scientist for the medical affairs team.

## Supporting information


**Figure S1.** Protein expression does not significantly differ between primary and metastatic tumors**.** Tumors from the primary and metastatic sites were compared for expression of proteins for which data were available for all four type of SCC. For ASCC *n* ≥ 25 (primary) and *n* ≥ 35 (metastatic). For CSCC *n* ≥ 42 (primary) and *n* ≥ 72 (metastatic). For OSCC *n* ≥ 22 (primary) and *n* ≥ 28 (metastatic). For VSCC *n* ≥ 15 (primary) and *n* ≥ 12 (metastatic).

**Table S1.** Probe and Threshold information for IHC and ISH

**Table S2.** P16 expression status of OSCC patient samples determined by IHC.

**Table S3.** Targeted sequencing results for patient samples tested.

**Table S4.** Summary of IHC, ISH, and sequencing results for each patient sample
