# Supplementary figures and images for: Comparative molecular profiling of HPV‐induced squamous cell carcinomas
Source: Cancer Med. 2017 May 29;6(7):1673–85. doi: 10.1002/cam4.1108 (PMC5504316; doi:10.1002/cam4.1108)

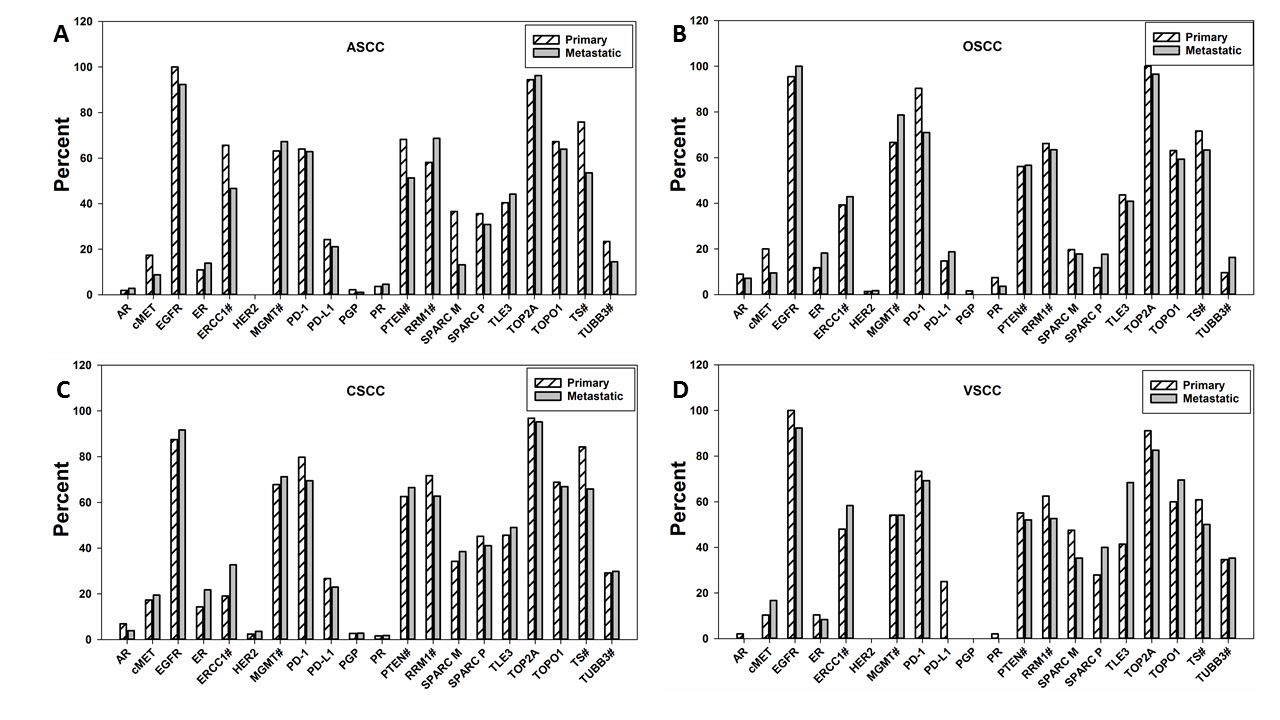

Supplement: Supplementary file 1 — Figure S1. Protein expression does not significantly differ between primary and metastatic tumors. Tumors from the primary and metastatic sites were compared for expression of proteins for which data were available for all four type of SCC. For ASCC n ≥ 25 (primary) and n ≥ 35 (metastatic). For CSCC n ≥ 42 (primary) and n ≥ 72 (metastatic). For OSCC n ≥ 22 (primary) and n ≥ 28 (metastatic). For VSCC n ≥ 15 (primary) and n ≥ 12 (metastatic). [file CAM4-6-1673-s001.tif]
