# Supplementary material for: Comparative molecular profiling of HPV‐induced squamous cell carcinomas
Source: Cancer Med. 2017 May 29;6(7):1673–85. doi: 10.1002/cam4.1108 (PMC5504316; doi:10.1002/cam4.1108)
Supplement: Supplementary file 2 — Table S1. Probe and Threshold information for IHC and ISH [file CAM4-6-1673-s002.docx]

| **Table S1. Probe and Threshold information for IHC and ISH** | | |
| --- | --- | --- |
| **Antibody (biomarker)** | **Clone or Probe** | **Threshold** |
| Anaplastic Lymphoma Kinase (ALK) | D53 | ≥3+ and ≥1% |
| Androgen receptor (AR) | AR27 | 0+ or <10% or ≥1+ and ≥10% |
| Breast cancer resistance protein (BCRP) | 6D171 | 0+ or <10% or ≥1+ and ≥10% |
| cKIT (CD117) | 9.7 | 0+ and =100% or ≥2+ and ≥30% |
| Hepatocyte growth factor recepotr (cMET) | SP44 | <50% or <2+ or ≥2+ and ≥50% |
| Cyclooxygenase-2 (COX2) |  | =0+ or <2+ and <10% or ≥2+ and ≥10% |
| Estrogen receptor (ER) | SP1 | 0+ or <10% or ≥1+ and ≥10% |
| Epidermal growth factor receptor (EGFR) | 31G7 | 2+ and ≥10% |
| Excision Repair Cross-Complementation group 1 (ERCC1) | 8F1 | <2+ or ≤3+ and <10% or =2+ and <50% or ≥3+ and ≥10% or ≥2+ and ≥50% |
| Human epidermal growth factor receptor 2 (HER2) | 4B5 | ≤1+ or =2+ and ≤10% or ≥3+ and >10% |
| 0(6)-methylguanine-methyltransferase (MGMT) | MT23.2 | 0+ or ≤35% or ≥1+ and >35% |
| Multidrug Resistance Protein 1 (MRP1) | 33A6 | 0+ or <10% or ≥1+ and ≥10% |
| Programmed cell death 1 (PD-1)* | NAT105 | =0+ or ≥1+ |
| Programmed cell death 1 ligand (PD-L1) | 130021/SP142 | <5% or <2+ or ≥2+ and ≥5% |
| Platelet-derived growth factor receptor A (PDGFRA) | 2D2-1A11 | =0+ and =100% or ≥2+ and ≥30% |
| P-glycoprotein (PGP) | C494 | 0+ or <10% or ≥1+ and ≥10% |
| Progesterone receptor (PR) | IE2 | 0+ or <10% or ≥1+ and ≥10% |
| Phosophatase and Tensin Homolog (PTEN) | 6H2.1 | 0+ or ≤50% or ≥1+ and >50% |
| Ribonucleotide reductase M1 (RRM1) | polyclonal | 0+ or <50% or <2+ or ≥2+ and ≥50% |
| Secreted protein, acidic, cysteine-rich (SPARC) | 122511 | <30% or <2+ or ≥2+ and ≥30% |
| Transducin-like enhancer of split 3 (TLE3) | polyclonal | <30% or <2+ or ≥2+ and ≥30% |
| Topoisomerase II alpha (TOP2A) | 3F6 | 0+ or <10% or ≥1+ and ≥10% |
| Topoisomerase I (TOPO1) | 1D6 | 0+ or <30% or <2+ or ≥2+ and ≥30% |
| Thymidylate synthase (TS) | TS106/4H4B1 | 0+ or ≤3+ and <10% or ≥1+ and ≥10% |
| Class III member of beta-tubulin (TUBB3) | polyclonal | <30% or <2+ or ≥2+ and ≥30% |
| cMET FISH/CISH | Probe | Positivity for increased gene copy number has been defined as ≥ 5 copies. |
| EGFR FISH/CISH | Probe | Positivity for increased gene copy number has been defined as ≥ 4 copies in 40% or more tumor cells. |
| HER2 FISH/CISH | Probe | HER2/Neu:CEP 17 signal ratio of ≥2.0 is amplified and <2.0 is not amplified per Abbott (Pathvysion), Herceptin and Ventana INFORM HER2 CISH package inserts. |
| PIK3CA FISH | Probe | PIK3CA:CEP3 signal ratio of ≥3.0 is amplified and <2.0 is not amplified |
| TOP2A FISH | Probe | TOP2A:CEP 17 signal ratio of ≥2.0 is amplified and <2.0 is not amplified |
